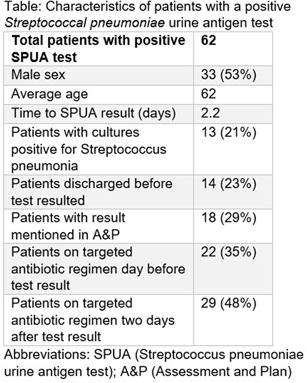# Impact of Streptococcus pneumoniae Urinary Antigen Testing in a Large Academic Medical Center

**DOI:** 10.1017/ash.2024.209

**Published:** 2024-09-16

**Authors:** Jensie Burton, Krutika Hornback, Rachel Burgoon, Ahmed M Albakheet

**Affiliations:** Medical University of South Carolina; MUSC Health; Student

## Abstract

**Background:** The Streptococcal pneumoniae urine antigen (SPUA) test was developed to increase microbiologic diagnosis of pneumonia. Concerns have been raised about the test’s low sensitivity and failure to alter outcomes by de-escalating antibiotics (PMID:31956656). However, the cost-effectiveness and real-world clinical utility of the test remain unclear. **Methods:** From June 1, 2022 - May 31, 2023, all patients with a SPUA test in the MUSC Health System were identified via Epic SlicerDicer. Those with a positive test underwent chart review. Antibiotics were classified as a “broad” or “targeted” regimen for S. pneumoniae. Targeted regimens included penicillins without beta-lactamase inhibitors, 1st-3rd generation cephalosporins, doxycycline, levofloxacin or moxifloxacin (with or without azithromycin), as well as azithromycin monotherapy. Broad regimens included 4th generation or higher cephalosporins, carbapenems, penicillins with beta-lactamase inhibitors, and vancomycin. **Results:** In one year, 1,518 patients had a SPUA test ordered. 62 (4%) patients had a positive test. Of those 62 patients, 14 patients were discharged before the test resulted (Table). The average turnaround time for the test was 2.2 days. When comparing antibiotic therapy on the day before the SPUA test resulted to two days after the test resulted, only 7 additional patients were switched to a targeted regimen (Figure). **Conclusion:** Of 1,518 SPUA tests ordered in a year, most (1,456 or 96%) were negative, with minimal changes to antibiotic therapy based on positive **Results:** These results are similar to other real-world studies, which showed a positive test prevalence between 4-8% (PMID:30265290) with 15-30% of patients changed to targeted antibiotics following a positive result (PMID:23111919, PMID: 28053969). The SPUA test cost approximately $44,022 (based on $29 test price) but has limited utility in a real-world setting.

**Disclosure:** Krutika Hornback: Speaker’s Bureau - Cepheid Diagnostics

**Figure:**
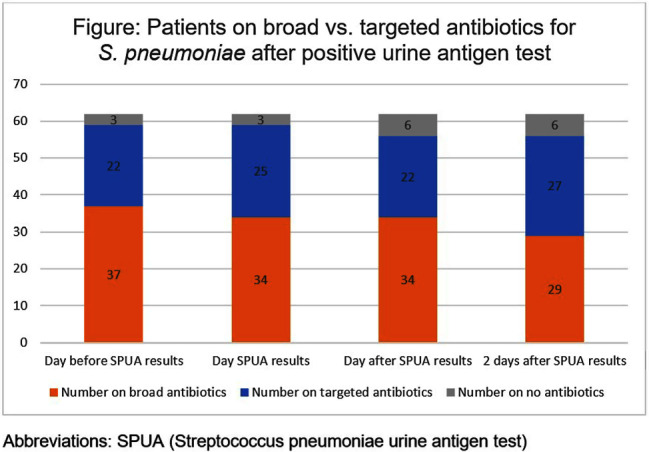

**Figure:**